# Enhancing colonic anastomotic healing with a novel melatonin‐loaded PLGA matrix

**DOI:** 10.1113/EP093987

**Published:** 2026-09-27

**Authors:** Ufuk Demir, Zekiye Gülfem Yurtgezen, Gülfem Erbil, Oruç Numan Gökçe, Ayhan Oral, Metehan Uzun

**Affiliations:** ^1^ Department of Physiology Faculty of Medicine, Çanakkale Onsekiz Mart University Çanakkale Türkiye; ^2^ Department of Histology and Embryology Faculty of Medicine, Çanakkale Onsekiz Mart University Çanakkale Türkiye; ^3^ Department of General Surgery Faculty of Medicine, Çanakkale Onsekiz Mart University Çanakkale Türkiye; ^4^ Department of Physico‐Chemistry Faculty of Science, Çanakkale Onsekiz Mart University Çanakkale Türkiye

**Keywords:** bursting pressure, colonic anastomosis, melatonin, nanofibre, wound healing

## Abstract

Anastomotic leakage continues to be a severe and prevalent complication in colorectal surgery, leading to elevated morbidity and mortality rates despite modern advancements in surgical and perioperative care. To address this persistent clinical challenge, the aim of the present study was to engineer a biocompatible poly(lactic‐co‐glycolic acid) (PLGA) matrix designed for the sustained release of melatonin, subsequently evaluating its therapeutic efficacy in promoting colon anastomotic repair. Seventy‐two male Wistar albino rats were randomly assigned to three experimental groups: anastomosis (*n* = 24); anastomosis + matrix (*n* = 24); and anastomosis + melatonin‐loaded matrix (*n* = 24). Anastomotic segments were harvested on postoperative days 3 and 7, and colonic bursting pressure was measured as a functional indicator of anastomotic integrity. The melatonin‐loaded matrix significantly enhanced anastomotic healing on postoperative day 7. Melatonin delivery: (1) markedly reduced the gene expression of pro‐inflammatory cytokines; (2) modulated *iNOS*, *COX‐2* and *MPO* expression on both days 3 and 7; (3) decreased *HO‐1* and catalase expression on day 7; (4) promoted collagen synthesis; (5) enhanced collagen deposition through upregulation of *Arg‐1*; and (6) regulated *ADAM10* and *ADAM17* gene expression. These results are correlated with the histological analysis findings. Collectively, controlled release of melatonin via a PLGA‐based biomaterial matrix promotes colon anastomosis healing. This strategy represents a promising translational approach for reducing postoperative anastomotic complications.

## Abstract (highlights)

- **What is the central question of this study?**
  Can sustained local delivery of melatonin using an electrospun poly(lactic‐co‐glycolic acid) (PLGA) matrix improve colonic anastomotic healing by enhancing mechanical strength and regulating the inflammatory and reparative processes involved in tissue repair?
- **What is the main finding and its importance?**
  A sustained‐release melatonin‐loaded PLGA matrix enhanced colonic anastomotic repair by improving mechanical integrity while modulating inflammation, oxidative stress, macrophage polarization and extracellular matrix remodelling. This biomaterial‐based local delivery strategy might represent a clinically translatable approach for improving postoperative anastomotic healing.

## 1 INTRODUCTION

Despite promising developments in colorectal surgical techniques, anastomotic leakage (AL) remains one of the most devastating postoperative complications (Fang et al., 2020). Reported incidence rates range from 2% to 19% in general colorectal procedures and can approach 30% in high‐risk settings, such as malignancy, obstruction or emergency surgery (Ma et al., 2025). Beyond early postoperative failure, AL can also present in the late phase, extending beyond 30 days, thereby substantially increasing hospital stay, healthcare costs, morbidity and mortality. Mortality rates associated with AL have been reported to reach 15%, and many affected patients require reoperation or permanent stoma formation (Shah et al., 2026; Botirov et al., 2022). These outcomes show that AL is still a serious ongoing problem in abdominal surgeries. Given these issues, understanding the biological mechanisms underlying anastomotic healing is of critical importance.

Anastomotic healing is not a singular pathological event but a coordinated biological process involving sequential phases of coagulation, inflammation, proliferation and remodelling (Su'a et al., 2017; Vallance et al., 2017). Disturbances in these phases can compromise anastomotic integrity through several interconnected mechanisms. In the early inflammatory phase, excessive activation of pro‐inflammatory cytokines, such as interleukin *(IL)‐6, IL‐1β* and tumor necrosis factor alpha (*TNF‐α*), together with increased activity of inflammatory mediators, including inducible nitric oxide synthase (iNOS), Cyclooxygenase‐2 (COX‐2) and myeloperoxidase (MPO), can amplify local tissue injury, leucocyte infiltration and oxidative damage (Reisinger et al., 2017; Stavely et al., 2023). Concurrent oxidative stress responses, reflected by alterations in enzymes such as Heme oxygenase‐1 (*HO‐1*) and *catalase*, can further impair cellular homeostasis in ischaemic or hypoxic conditions (Pantelis et al., 2011; Paul et al., 2005). As healing progresses, inadequate activation of reparative signalling pathways involving *TGF‐β1* can disrupt fibroblast activity and extracellular matrix production, leading to insufficient synthesis of structural collagen components, such as collagen type I alpha 1 chain (COL1A1) and collagen type III alpha 1 chain (COL3A1), which are essential for restoring tensile strength (Morgan & Shogan, 2022). In parallel, dysregulation of macrophage polarization markers, such as Arginase‐1 (Arg‐1), can prolong inflammatory dominance rather than allowing a transition towards tissue repair (Cira et al., 2025; Ge et al., 2026). Finally, imbalanced activity of matrix‐regulating enzymes, including ADAM metallopeptidase domain 10 (*ADAM10*) and ADAM metallopeptidase domain 17 (*ADAM17*), can alter extracellular matrix remodelling and cell–matrix interactions. This disruption can affect collagen organization, angiogenesis and fibroblast proliferation, processes that collectively determine the structural stability of the anastomosis (Ågren & Auf Dem Keller, 2020; Sikora‐Skrabaka et al., 2022).

Multiple reinforcement strategies have been investigated to improve anastomotic stability and reduce the incidence of AL. Among emerging approaches, polymer‐based delivery systems have attracted considerable attention because they can provide both mechanical support and localized therapeutic effects (Ho & Ashour, 2010; Slieker et al., 2013). Poly(lactic‐co‐glycolic acid) (PLGA) is widely used in surgical biomaterials owing to its biocompatibility, predictable degradation kinetics and ability to form stable matrices that gradually release incorporated agents (Luchtefeld et al., 2022). Within the pathological environment of anastomotic healing, melatonin represents a promising therapeutic molecule owing to its potent antioxidant and anti‐inflammatory properties, in addition to its ability to enhance fibroblast activity, collagen deposition and angiogenesis (Guclu et al., 2014; Özkan et al., 2018). However, its oral bioavailability is relatively low owing to rapid metabolism and first‐pass hepatic degradation, which limits the amount of active compound reaching target tissues. Therefore, controlled and localized delivery systems might substantially enhance its therapeutic efficacy by maintaining sustained concentrations at the injury site while minimizing systemic loss (Kulka‐Kamińska et al., 2025; Mirza‐Aghazadeh‐Attari et al., 2022). In this context, incorporation of melatonin into a controlled‐release matrix offers a rational strategy to prolong its biological activity and optimize its regenerative effects during tissue healing. Therefore, a melatonin‐loaded PLGA matrix might provide a dual therapeutic advantage in this setting by mechanically reinforcing the anastomotic line while simultaneously modulating inflammatory, oxidative and extracellular matrix‐related pathways that are crucial for successful tissue repair.

Although previous experimental studies have demonstrated the beneficial effects of melatonin on wound and intestinal healing, these investigations have focused primarily on systemic administration or conventional local application. Likewise, degradable biomaterials have been explored as mechanical reinforcement strategies for colorectal anastomoses, yet studies integrating sustained local melatonin delivery with a degradable electrospun PLGA matrix remain lacking. Therefore, whether controlled local release of melatonin can simultaneously provide mechanical reinforcement and sustained biological modulation of inflammatory, oxidative stress, extracellular matrix remodelling and angiogenic processes during colon anastomotic healing remains unknown. The present study was designed to address this important knowledge gap. Accordingly, the aim of the present study was to evaluate the therapeutic efficacy of a melatonin‐loaded electrospun PLGA nanofibrous matrix on colon anastomotic healing by assessing functional, histological and molecular outcomes associated with tissue repair.

## 2 MATERIALS AND METHODS

### 2.1 In vitro fabrication and characterization of PLGA–PEG200 nanofibres

A polymer blend was prepared as previously described (Gökçe et al., 2024). Briefly, poly(lactic‐co‐glycolic acid) (PLGA; lactide:glycolide ratio 70:30, ester‐terminated; Sigma‐Aldrich, St. Louis, MO, USA) and polyethylene glycol 200 (PEG200, purity ≥99%; Merck, Darmstadt, Germany) were used to prepare a polymer solution with a total polymer concentration of 15% (w/w) at a PLGA:PEG200 ratio of 70:30 (w/w). PLGA was initially dissolved in a dimethylformamide–dichloromethane solvent mixture (70:30, w/w), using analytical‐grade dimethylformamide (Merck) and HPLC‐grade dichloromethane (Merck), followed by the addition of PEG200. The solution was magnetically stirred at room temperature for 1 h until homogeneous. Nanofibres were produced by electrospinning using the following parameters: 16 kV applied voltage; 17 cm tip‐to‐collector distance; and 0.85 mL h^−1^ flow rate.

#### 2.1.1 Preparation of melatonin‐loaded PLGA–PEG200 nanofibres

Melatonin‐loaded nanofibres were produced using the method mentioned above; melatonin (purity ≥98%; Sigma‐Aldrich, St. Louis, MO, USA) was added to the homogeneous polymer solution at the final stage to achieve a loading of 1 mg cm^−2^ (total 176 mg melatonin; dosing calculated as 1 cm × 1 cm nanofibre/1 mg melatonin). After the addition, the mixture was stirred for ≥1 h until homogeneous and electrospun using the same parameters. This loading concentration was selected based on our preliminary formulation studies demonstrating homogeneous fibre formation and sustained release characteristics, together with previously published studies reporting the biological activity of locally delivered melatonin (Aykora et al., 2025; Gökçe et al., 2024).

#### 2.1.2 In vitro degradation assay

Nanofibre specimens (∼1 cm × 3 cm) were cut and weighed to obtain baseline mass, then incubated in 10 mL PBS (0.1 M, pH 7.4) at 37°C for 7 days. Every 24 h, samples were removed, dried in a vacuum oven for 24 h, reweighed, and the mass loss (as a percentage) was calculated. Mass loss–time profiles were generated (Gökçe et al., 2024).

#### 2.1.3 In vitro melatonin release profile

For release testing, nanofibre samples (∼20 mg, *n* = 3; excluding PLGA‐only fibres) were placed into a dialysis membrane, supplemented with 1 mL PBS (pH 7.4) and immersed in 20 mL PBS (pH 7.4). The absorbance maxima for melatonin were determined by ultraviolet scanning (190–300 nm), and calibration curves were generated using six standard concentrations in PBS at the identified wavelengths (220 and 280 nm). Release was quantified in PBS (pH ∼7) at predefined time points (hourly for the first 6 h, then once daily) (Zhang et al., 2013).

#### 2.1.4 Morphological and chemical characterization

Surface morphology and fibre architecture were assessed by scanning electron microscopy (Zeiss Gemini‐500 SEM‐EDX), evaluating fibre continuity, distribution, diameter, porosity/roughness and homogeneity. Energy‐dispersive X‐ray spectroscopy was used to support the presence/distribution of melatonin within the polymer matrix. Samples were rendered conductive via coating before imaging. The chemical composition was also examined using Fourier‐transform infrared spectroscopy (FTIR; PerkinElmer Spectrum One; 400–4000 cm^−1^); melatonin presence was inferred from characteristic N–H peaks (Altindal & Gumusderelioglu, 2016).

#### 2.1.5 Sterilization and storage

Prior to implantation, the electrospun mats were trimmed into 2 cm × 2 cm sterile‐ready patches and subjected to ultraviolet irradiation for 30 min to achieve surface decontamination. Following sterilization, each patch was handled in aseptic conditions, individually vacuum‐sealed in sterile nylon pouches to minimize recontamination and moisture exposure, and stored at 4°C until surgery to preserve structural integrity and ensure consistent handling characteristics at the time of application (Romeo et al., 2022).

### 2.2 In vivo experimental design

#### 2.2.1 Animals

A total of 72 male Wistar rats (2–3 months old, 200–350 g) were obtained from Çanakkale Onsekiz Mart University Experimental Research Centre (ÇOMÜDAM) and maintained at 21°C ± 2°C, 50% humidity, under a 12 h–12 h light–dark cycle, with ad libitum access to food and water. All experimental procedures were conducted in accordance with institutional and national guidelines for the care and use of laboratory animals and were approved by the Çanakkale Onsekiz Mart University Animal Experiments Local Ethics Committee (ÇOMÜ‐HADYEK) with the decision number 2023/11‐05. The sample size was determined by an a priori power analysis performed using G*Power software (v.3.1.9.7) before the initiation of the study. The calculation was based on the primary outcome measure (colonic bursting pressure; Karliczek et al., 2008), using a one‐way ANOVA (fixed effects, omnibus test), with a significance level (α) of 0.05, a statistical power (1 − β) of 0.80, and an anticipated large effect size. The analysis indicated that a minimum of 12 animals per experimental group was required at each postoperative time point. Additional reserve animals were included at the beginning of the study to compensate for anticipated perioperative mortality and ensure that the predetermined sample size required by the power analysis was achieved. A total of 72 animals completed the study and were included in the final analyses.

#### 2.2.2 Experimental groups

Animals were randomized by body weight into three main groups, each subdivided into postoperative day (POD) 3 and 7 cohorts (*n* = 12 per subgroup; total six subgroups): anastomosis (A; *n* = 24), colon anastomosis only; anastomosis + matrix (A+Matrix; *n* = 24), anastomosis with melatonin‐free 2 cm × 2 cm matrix; and anastomosis + matrix + melatonin (A+Matrix+Mel; *n* = 24), anastomosis plus melatonin‐loaded 2 cm × 2 cm matrix. At each time point, six rats per group were used for bursting pressure, gene expression and western blot analyses, and six rats per group for histopathology.

#### 2.2.3 Colon anastomosis model and matrix placement

After 12 h fasting, general anaesthesia was induced with ketamine (70 mg kg^−1^; Ketalar®, Pfizer, USA) + xylazine (7 mg kg^−1^; Rompun®, Bayer, Germany), intramuscularly (Erbil & Uzun, 2024). Following abdominal shaving and 10% povidone–iodine preparation, a midline laparotomy was performed in aseptic conditions. The left colon was exteriorized and transected near the colorectal junction, and an end‐to‐end, single‐layer anastomosis was created using 6/0 polypropylene (Prolene). The fascia was closed with simple sutures, and the skin with continuous sutures using 3/0 Prolene. To prevent fluid loss, 5 mL of warmed saline was administered subcutaneously intraoperatively. In matrix‐treated groups, 2 cm × 2 cm nanofibre sheets were wrapped circumferentially around the anastomotic line and fixed with minimal suturing to prevent slippage (melatonin‐loaded matrix in A+Matrix+Mel, melatonin‐free matrix in A+Matrix).

#### 2.2.4 Tissue harvesting

On POD 3 and POD 7, rats were euthanized via cervical dislocation under ketamine/xylazine (7/70 mg kg^−1^) general anaesthesia. A colon segment including the anastomosis with 2 cm proximal and 2 cm distal margins was resected and kept in 4°C saline for measurement of bursting pressure. After bursting pressure testing, a smaller segment including 0.5 cm proximal and 0.5 cm distal to the anastomosis was collected for qRT‐PCR and western blot and stored at −80°C. Separate specimens for histology were fixed in 10% formaldehyde.

#### 2.2.5 Bursting pressure

Resected colon segments were rinsed with saline to remove faecal material. One end was connected to an infusion pump, the other to a pressure transducer integrated with a data acquisition system (Biopac MP35) and secured leak‐proof using 2/0 silk. Infusion of 4°C saline was delivered at 4 mL min^−1^ (Harvard Apparatus) while pressure was recorded continuously. The maximum pressure rupture at the anastomotic site was defined as the bursting pressure. Measurements were performed in a container filled with cooled saline to minimize biochemical alteration before downstream analyses (Pehlivanlı et al., 2018).

#### 2.2.6 Tissue homogenization and protein isolation for Western blot

Colon tissues were homogenized mechanically using a TissueLyser LT with tungsten carbide beads (two) and micro glass beads (20), applying three cycles of high‐frequency disruption (1 min each) with interim cooling steps. For protein extraction, 20–50 mg of tissue was lysed in RIPA buffer supplemented with phenylmethylsulphonyl fluoride, sodium orthovanadate and protease inhibitor, homogenized (27 000 rpm) in cold conditions, and centrifuged (14 000*g*, 20 min). Protein concentration was determined by A280 using a microvolume spectrophotometer. Samples were standardized, pooled within groups, denatured at 80°C for 12 min, separated on 10% Bis–Tris gels, and transferred using iBlot 2. Membranes were blocked and incubated overnight at 4°C with primary antibodies against iNOS, COL1A1, COL3A1, CD86 (B7‐2), CD206 (MMR) and β‐actin, followed by appropriate secondary antibodies. Bands were visualized using Micro ChemiDoc and quantified by densitometry, normalized to β‐actin and expressed as relative intensity versus control (Özbey & Uzun, 2024).

#### 2.2.7 RNA isolation, complementary DNA synthesis and qRT‐PCR

Total RNA was isolated from tissue using a kit‐based protocol combined with TRIzol, quantified for purity, equalized, and stored at −80°C. Complementary DNA was synthesized using kit reagents with input RNA standardized to 600 ng mL^−1^ in the following cycling conditions: 25°C (10 min), 37°C (120 min), 85°C (5 min) and 4°C (hold). Gene expression was assessed by qRT‐PCR (Applied Biosystems QuantStudio 5) for *VEGF*, *b‐FGF*, *TGF‐β1*, *TNF‐α*, *IL‐1β*, *IL‐6*, *IL‐10*, *iNOS*, *MPO*, *CAT*, *α‐SMA*, *COX‐2*, *Arg‐1*, *ADAM10*, *ADAM17*, *HO‐1*, *MMP2*, *MMP9*, *COL1A1* and *COL3A1* using gene‐specific primers listed in the manuscript. Cycling conditions were as follows: 95°C (3 min), followed by repeated cycles of 95°C (15 s) and 60°C (1 min), with melt‐curve analysis (95°C for 15 s, 60°C for 1 min and 95°C for 15 s). Relative expression was calculated using the 2^−^^ΔΔ^ *^Ct^* method, normalized to 18S rRNA and *GAPDH*.

#### 2.2.8 Histopathology

Histological evaluation was performed by a blinded researcher using colon tissues (six animals per group per time point). Specimens were fixed in 10% formaldehyde, processed routinely, embedded in paraffin, and sectioned at 5 µm thickness. Sections were stained with Haematoxylin and Eosin for general morphology and scored using an evaluation table adapted from Ehrlich–Hunt and Verhofstad systems [necrosis, polymorphonuclear leucocyte (PMNL) and mononuclear leucocyte infiltration, oedema, mucosal epithelium, granulation, neoangiogenesis, fibroblasts and collagen]. Masson's Trichrome staining was used to evaluate collagen deposition (Castro et al., 2006; Yurtgezen et al., 2026).

### 2.3 Statistical analysis

Data were analysed using SPSS Statistics software (v.20.0). Descriptive statistics are presented as the mean ± SD. Normality of the data distribution was verified using the Shapiro–Wilk test. Differences among the experimental groups were evaluated using one‐way ANOVA. When a significant effect was detected, Tukey's honest significant difference (HSD) *post hoc* test was performed for pairwise comparisons. Differences were considered statistically significant at *P* ≤ 0.05. Exact *P*‐values are provided for all comparisons to ensure transparency.

## 3 RESULTS

### 3.1 Nanofibre fabrication and physicochemical characterization

Electrospun PLGA–PEG200 nanofibrous matrices were successfully fabricated and subsequently characterized to determine their degradation behaviour, drug release profile and structural properties.

**FIGURE 1:**
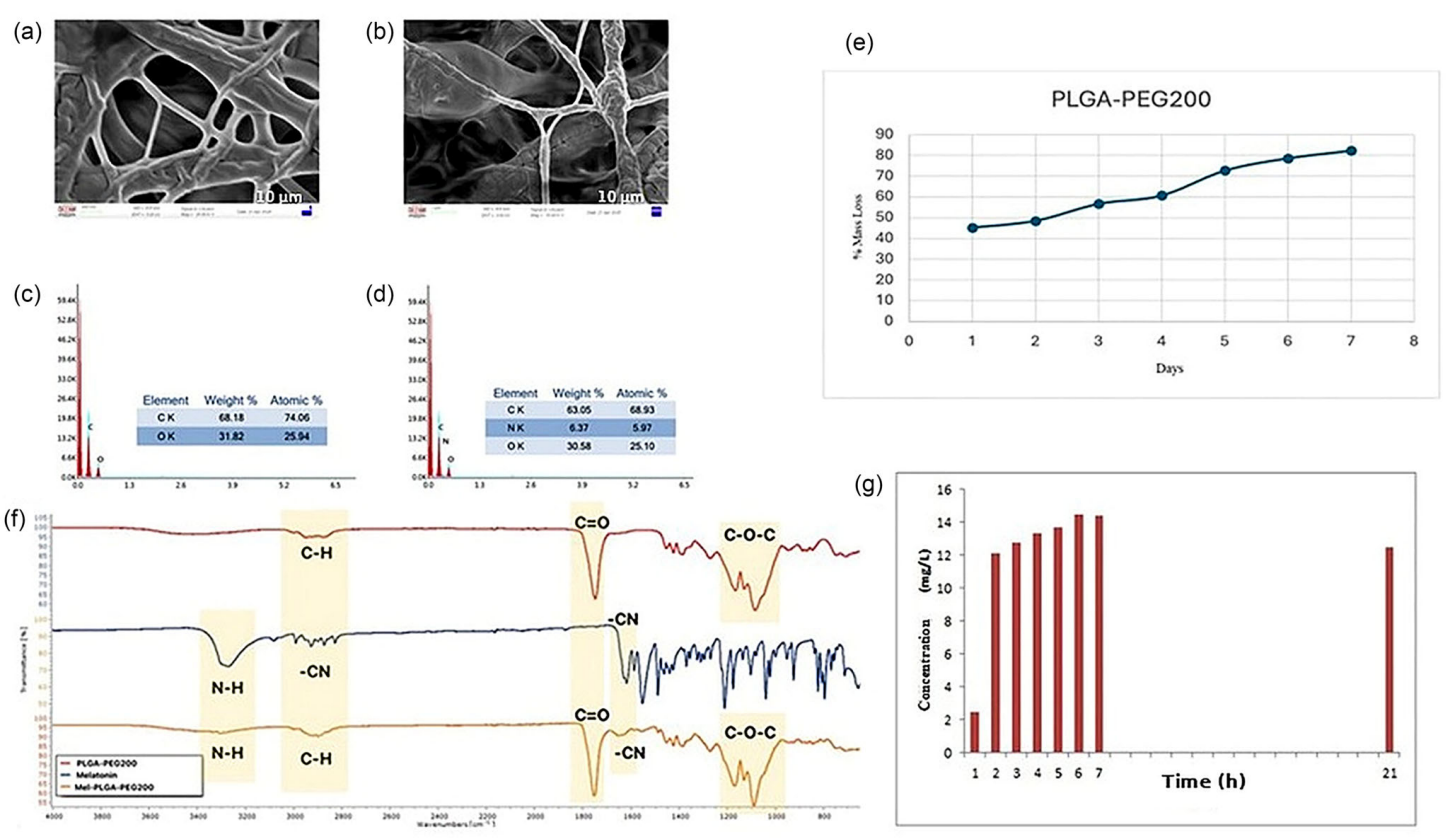
Morphological and physicochemical characterization of the electrospun nanofibres. (a) SEM micrograph of the pristine PLGA–PEG200 nanofibres (scale bar represents 10 µm). (b) SEM micrograph of the melatonin‐loaded PLGA–PEG200 (Mel–PLGA–PEG200) nanofibres (scale bar represents 10 µm). (c) Energy‐dispersive X‐ray spectrum and elemental composition (weight and atomic percentages) of the PLGA‐PEG200 scaffold. (d) Energy‐dispersive X‐ray spectrum of the Mel–PLGA–PEG200 scaffold, indicating the successful incorporation of melatonin through emergence of the nitrogen (N) peak. (e) In vitro degradation profile of the PLGA–PEG200 scaffold, represented as the percentage of mass loss over 7 days. (f) Fourier transform infrared (FTIR) spectra of the PLGA–PEG200 scaffold, pure melatonin and the Mel–PLGA–PEG200 scaffold, confirming drug encapsulation via the presence of characteristic stretching vibrations (N–H, C–H, –CN, C = O, C–O–C). (g) In vitro release profile of melatonin from the nanofibrous scaffold, showing the drug concentration (in milligrams per litre) over time (in hours). Abbreviations: C–H, carbon–hydrogen; C=O, carbonyl; C–O–C, ester/ether linkage; –CN, nitrile; EDX, energy‐dispersive X‐ray; FTIR, Fourier transform infrared; h, hour; Mel, melatonin; N, nitrogen; N–H, nitrogen–hydrogen; PLGA, poly(lactic‐co‐glycolic acid); PEG, polyethylene glycol; SEM, scanning electron microscopy.

The surface morphology of the nanofibres was examined by scanning electron microscopy. Both melatonin‐loaded and unloaded PLGA–PEG nanofibre scaffolds displayed randomly oriented, interwoven fibre networks with irregular yet continuous fibrous architecture, characteristic of electrospun polymer matrices. No major structural collapse or aggregation was observed, indicating that incorporation of melatonin did not disrupt fibre formation (Figure 1a,b).

To confirm melatonin incorporation, energy‐dispersive X‐ray spectroscopy analysis was performed. In the melatonin‐free PLGA–PEG nanofibre samples, the elemental composition consisted primarily of carbon (68.18%) and oxygen (31.82%), consistent with the expected polymer composition. In contrast, the melatonin‐loaded nanofibres showed 63.05% carbon, 30.58% oxygen and 6.37% nitrogen. The presence of nitrogen exclusively in the melatonin‐loaded samples confirmed the successful incorporation of melatonin within the nanofibrous matrix (Figure 1c,d).

In the degradation analysis, nanofibre samples (∼1 cm × 3 cm) were incubated in physiological conditions to evaluate their mass loss over time. The matrix was designed to support sustained melatonin release over ∼7 days, corresponding to the critical early phase of anastomotic healing. Consistent with this design objective, the nanofibre scaffold exhibited progressive degradation and lost ∼80% of its initial mass within 7 days, indicating a degradation profile compatible with short‐term therapeutic delivery (Figure 1e).

Chemical characterization was also conducted using FTIR. The FTIR spectrum of the PLGA–PEG nanofibres displayed characteristic peaks corresponding to both polymers. A prominent band at ∼1750 cm^−1^ corresponded to the C = O stretching vibration of ester bonds in PLGA, which is a characteristic absorption band of the aliphatic polyester backbone of PLGA and is consistent with previously reported PLGA FTIR spectra (Kumari et al., 2010; Makadia & Siegel, 2011). Peaks observed around 1080–1100 cm^−1^ were attributed to C–O–C stretching vibrations of PEG ether linkages, which represent the characteristic functional groups of PEG chains. Similar PEG‐associated absorption bands have been reported previously in PEG‐containing polymeric systems (Danhier et al., 2012). Signals between 1300 and 1400 cm^−1^ were assigned to C–H bending vibrations of methyl groups originating from the lactide and glycolide units of PLGA. Additionally, bands between 2800 and 2900 cm^−1^ corresponded to stretching vibrations of CH, CH_2_ and CH_3_ groups, which are characteristic of the aliphatic hydrocarbon structures present in PLGA and PEG chains (Figure 1f). These spectral features are in agreement with previously reported FTIR profiles of PLGA–PEG‐based materials (Danhier et al., 2012; Makadia & Siegel, 2011). The FTIR spectrum of melatonin exhibited its own characteristic signals, including a band around 3300 cm^−1^ corresponding to N–H stretching, peaks between 2800 and 3000 cm^−1^ related to ‐CN group stretching, and a band near 1600 cm^−1^ associated with secondary amide vibrations. In the spectrum of melatonin‐loaded PLGA–PEG nanofibres, characteristic peaks corresponding to both the polymer matrix and melatonin were detected. However, the melatonin‐associated bands appeared less intense, which is consistent with the relatively low proportion of melatonin within the composite nanofibre formulation. The absence of significant peak shifts or the appearance of new bands suggests that melatonin was physically incorporated into the PLGA–PEG nanofibre matrix without inducing major chemical modifications of the polymer structure.

The in vitro melatonin release profile demonstrated an initial rapid increase in melatonin concentration during the early phase of incubation. The melatonin concentration in the surrounding medium reached ∼2.5 mg mL^−1^ at 1 h, followed by a sharp increase to 12 mg mL^−1^ at 2 h. Subsequent measurements showed a gradual increase to 12.5 mg mL^−1^ at 3 h, 13 mg mL^−1^ at 4 h, 13.5 mg mL^−1^ at 5 h and 14 mg mL^−1^ at 6 h. After this phase, the release rate slowed and approached a plateau, with the melatonin concentration stabilizing at ∼12.5 mg mL^−1^ by the 21st hour. Overall, the release kinetics revealed an initial burst phase (0–2 h) followed by a slower release phase (3–6 h) and eventual stabilization, suggesting that the electrospun matrix provides early drug availability followed by sustained exposure (Figure 1g).

### 3.2 In vivo findings

During the experimental period, 14 animals died in the perioperative period and were excluded from the analyses. Reserve animals were used to maintain the predetermined sample size, and data from 72 animals were included in the final analyses. Macroscopic anastomotic leakage was observed in eight animals, generalized peritonitis in four animals, and two animals died because of anaesthesia‐related complications before postoperative assessment. No intra‐abdominal abscesses were observed.

Baseline body weights were recorded on day 0 for all animals, and pre‐euthanasia weights were documented at the scheduled end points (POD 3 and POD 7). Across groups, body weights showed only modest perioperative variation, with no pattern suggestive of group‐specific morbidity.

After euthanasia on days 3 and 7, the resected colon segments were used to test *ex vivo* bursting pressure, followed by molecular (qRT‐PCR and western blot) and histopathological assessments.

### 3.3 *Ex vivo* bursting pressure

Bursting pressure was measured on colon segments containing the anastomosis using a pressure transducer connected to the Biopac MP35 system. The peak of the pressure curve immediately before rupture was defined as the bursting pressure and expressed in millimetres of mercury. On POD 3, bursting pressure values were comparable across the A, A+Matrix and A+Matrix+Mel groups, and no statistically significant differences were detected. On POD 7, the A+Matrix+Mel group demonstrated a marked improvement in anastomotic strength. The bursting pressure in the A+Matrix+Mel group (253.78 ± 29.75 mmHg) was significantly higher than in both A (150.09 ± 15.70 mmHg) and A+Matrix (163.00 ± 24.12 mmHg) (*P* = 0.014 and *P* = 0.045, respectively). In contrast, bursting pressure did not differ significantly between A and A+Matrix groups at day 7.

### 3.4 Molecular analyses

#### 3.4.1 qRT‐PCR gene expression profiling

Gene expression was quantified at days 3 and 7 in anastomotic colon tissues for the following targets: *IL‐1β*, *IL‐6*, *TNF‐α*, *iNOS*, *COX‐2*, *MPO*, *HO‐1*, *CAT*, *VEGF*, *b‐FGF*, *TGF‐β1*, *COL1A1*, *COL3A1*, *α‐SMA*, *Arg‐1*, *MMP2*, *MMP9*, *ADAM10* and *ADAM17*.

#### 3.4.2 Pro‐inflammatory cytokine and effector enzyme profiles

**FIGURE 2:**
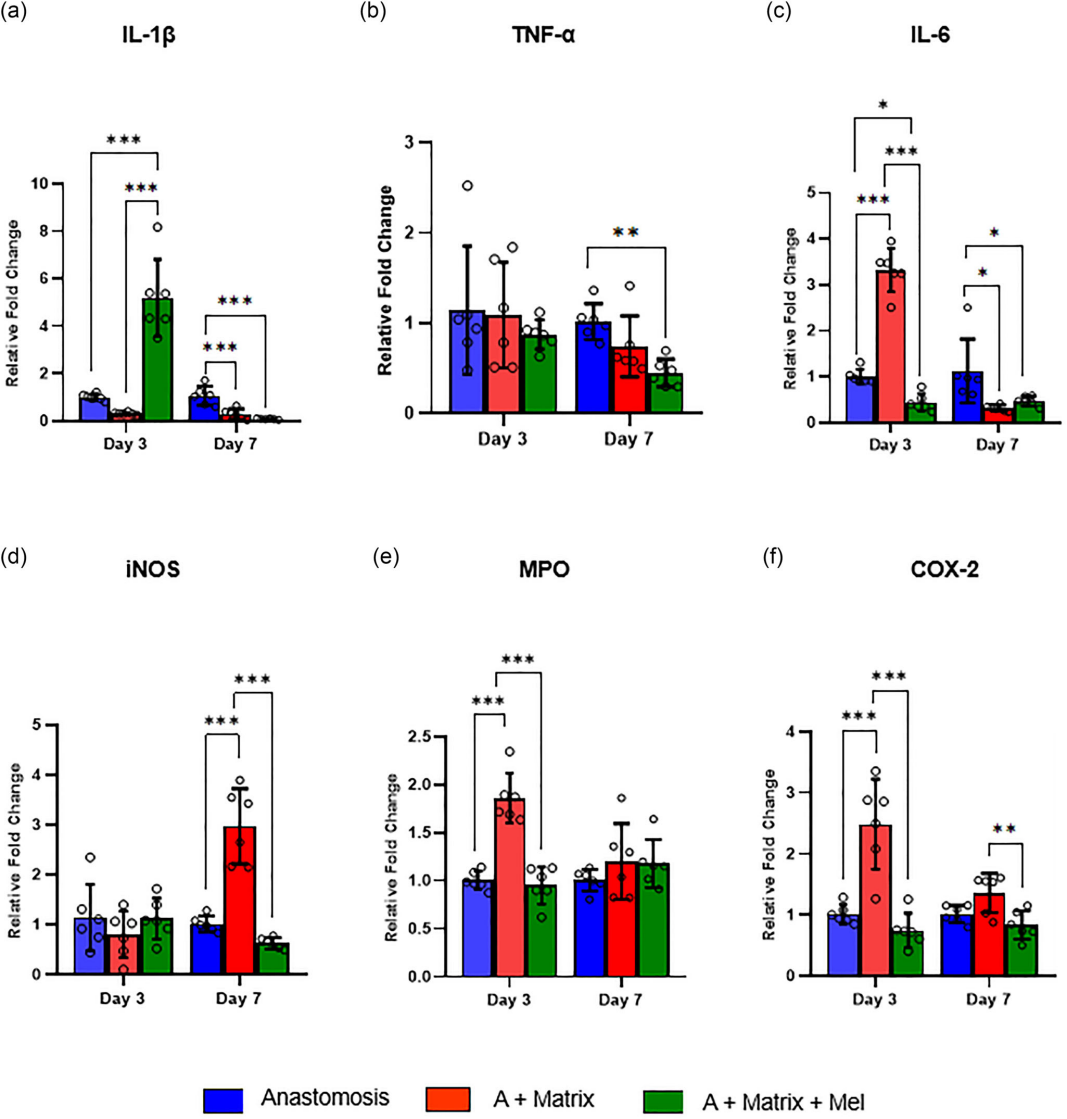
Relative mRNA expression of pro‐inflammatory cytokines and inflammatory enzymes in colonic anastomotic tissues at postoperative days 3 and 7. Bar graphs with overlaid individual data points represent the relative mRNA expression of the following: (a) *IL‐1β*; (b) *TNF‐α*; (c) *IL‐6*; (d) *iNOS*; (e) *MPO*; and (f) *COX‐2*. Data are presented as the mean ± SD (*n* = 6 animals per experimental group at each time point). Asterisks indicate statistically significant differences between the indicated experimental groups (*P ≤ 0.05, **P ≤ 0.01 and ***P ≤ 0.001).

The expression of pro‐inflammatory cytokines and inflammatory enzymes differed among the experimental groups at PODs 3 and 7 (Figure 2a–f).

At POD 3, *IL‐1β* expression was significantly higher in the A+Matrix+Mel group than in both the A (*P* = 0.000004) and A+Matrix groups (*P* = 0.000001), whereas no significant difference was detected between the A and A+Matrix groups (*P* = 0.432). By POD 7, *IL‐1β* expression decreased markedly in all groups; however, the A+Matrix+Mel group exhibited significantly lower expression than the A group (*P* = 0.000032), whereas no significant difference was observed between the A+Matrix and A+Matrix+Mel groups (*P* = 0.345).

No significant differences in *TNF‐α* expression were observed among the experimental groups at POD 3. At POD 7, *TNF‐α* expression was significantly lower in the A+Matrix+Mel group than in the A group (*P* = 0.003), whereas the remaining pairwise comparisons were not statistically significant (*P* = 0.159 for A vs. A+Matrix and *P* = 0.125 for A+Matrix vs. A+Matrix+Mel).

For *IL‐6*, the A+Matrix group demonstrated the highest expression at POD 3, differing significantly from both the A (*P* < 0.0001) and A+Matrix+Mel (*P* < 0.0001) groups. Additionally, the A+Matrix+Mel group showed significantly lower expression than the A group (*P* = 0.016). At POD 7, *IL‐6* expression was significantly higher in the A group than in both the A+Matrix (*P* = 0.010) and A+Matrix+Mel (*P* = 0.038) groups, and no significant difference was observed between the A+Matrix and A+Matrix+Mel groups (*P* = 0.782).

No significant differences in *iNOS* expression were detected at POD 3. In contrast, at POD 7, the A+Matrix group exhibited significantly higher *iNOS* expression than both the A (*P* = 0.000005) and A+Matrix+Mel (*P* = 0.000001) groups, whereas the latter two groups did not differ significantly (*P* = 0.322).

Likewise, *MPO* expression was significantly higher in the A+Matrix group than in both the A (*P* = 000004) and A+Matrix+Mel (*P* = 0.000002) groups at POD 3. By POD 7, *MPO* expression was comparable among all experimental groups, with no statistically significant differences detected (A vs. A+Matrix, *P* = 0.465; A vs. A+Matrix+Mel, *P* = 0.537; A+Matrix vs. A+Matrix+Mel, *P* = 0.991).

For *COX‐2*, the A+Matrix group demonstrated significantly higher expression than both the A (*P* = 0.000167) and A+Matrix+Mel (*P* = 0.000028) groups at POD 3. At POD 7, *COX‐2* expression was significantly higher in the A+Matrix group than in the A+Matrix+Mel group (*P* = 0.005), whereas no significant differences were observed between the A group and the other experimental groups (A vs. A+Matrix, *P* = 0.061; A vs. A+Matrix+Mel, *P* = 0.443).

#### 3.4.3 Oxidative stress and antioxidant defence mechanisms

**FIGURE 3:**
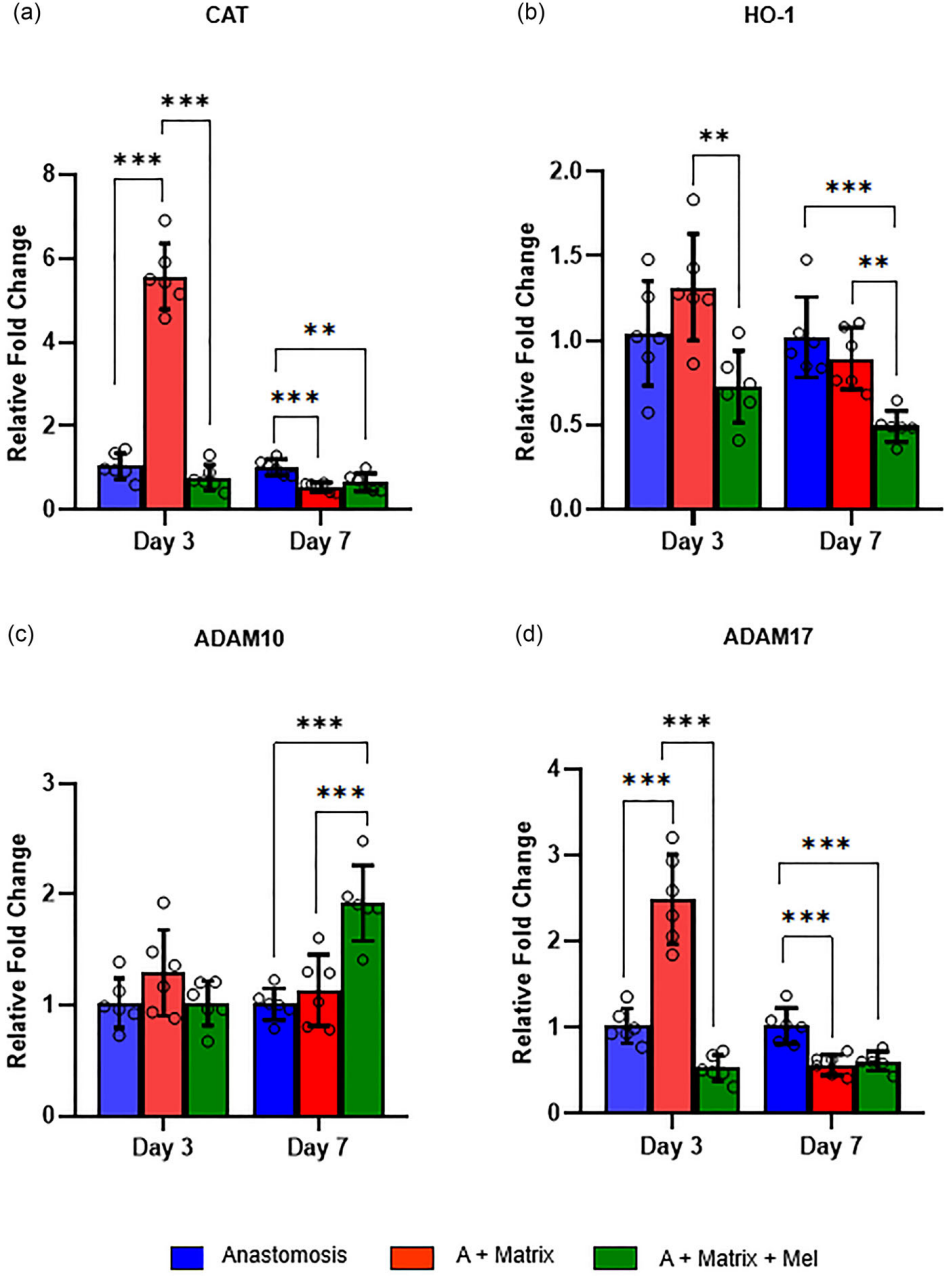
Relative mRNA expression of oxidative stress‐related genes and ADAM family proteases in colonic anastomotic tissues at postoperative days 3 and 7. Bar graphs with overlaid individual data points represent the relative mRNA expression of the following: (a) *CAT*; (b) *HO‐1*; (c) *ADAM10*; and (d) *ADAM17*. Data are presented as the mean ± SD (*n* = 6 animals per experimental group at each time point). Asterisks indicate statistically significant differences between the indicated experimental groups (*P ≤ 0.05, **P ≤ 0.01 and ***P ≤ 0.001).

The expression of antioxidant enzymes and ADAM family proteases differed among the experimental groups during the healing process (Figure 3a–d).

At POD 3, *CAT* expression was significantly higher in the A+Matrix group than in both the A (*P* < 0.0001) and A+Matrix+Mel (*P* < 0.0001) groups. By POD 7, *CAT* expression markedly declined in the A+Matrix group; both the A (*P* = 0.001) and A+Matrix+Mel (*P* = 0.009) groups exhibited significantly higher *CAT* expression compared with the A+Matrix group, whereas no significant difference was observed between the A+Matrix and A+Matrix+Mel groups (*P* = 0.540).

For *HO‐1*, the A+Matrix+Mel group exhibited significantly lower expression than the A+Matrix group at POD 3 (*P* = 0.007). At POD 7, *HO‐1* expression remained significantly lower in the A+Matrix+Mel group than in both the A (*P* = 0.0004) and A+Matrix (*P* = 0.004) groups, whereas no significant difference was observed between the A and A+Matrix groups (*P* = 0.464).

No significant differences in *ADAM10* expression were detected among the experimental groups at POD 3. In contrast, at POD 7, the A+Matrix+Mel group demonstrated significantly higher *ADAM10* expression than both the A (*P* = 0.00015) and A+Matrix (*P* = 00065) groups, whereas no significant difference was detected between the A and A+Matrix groups (*P* = 0.725).

For *ADAM17*, the A+Matrix group exhibited significantly higher expression than both the A (*P* = 0.000005) and A+Matrix+Mel (*P* < 0.0001) groups at POD 3. By POD 7, *ADAM17* expression was significantly higher in the A group than in both the A+Matrix (*P* = 0.0003) and A+Matrix+Mel (*P* = 0.0008) groups, whereas no significant difference was detected between the A+Matrix and A+Matrix+Mel groups (*P* = 0.880).

### 3.5 Angiogenesis, proliferation and macrophage polarization

**FIGURE 4:**
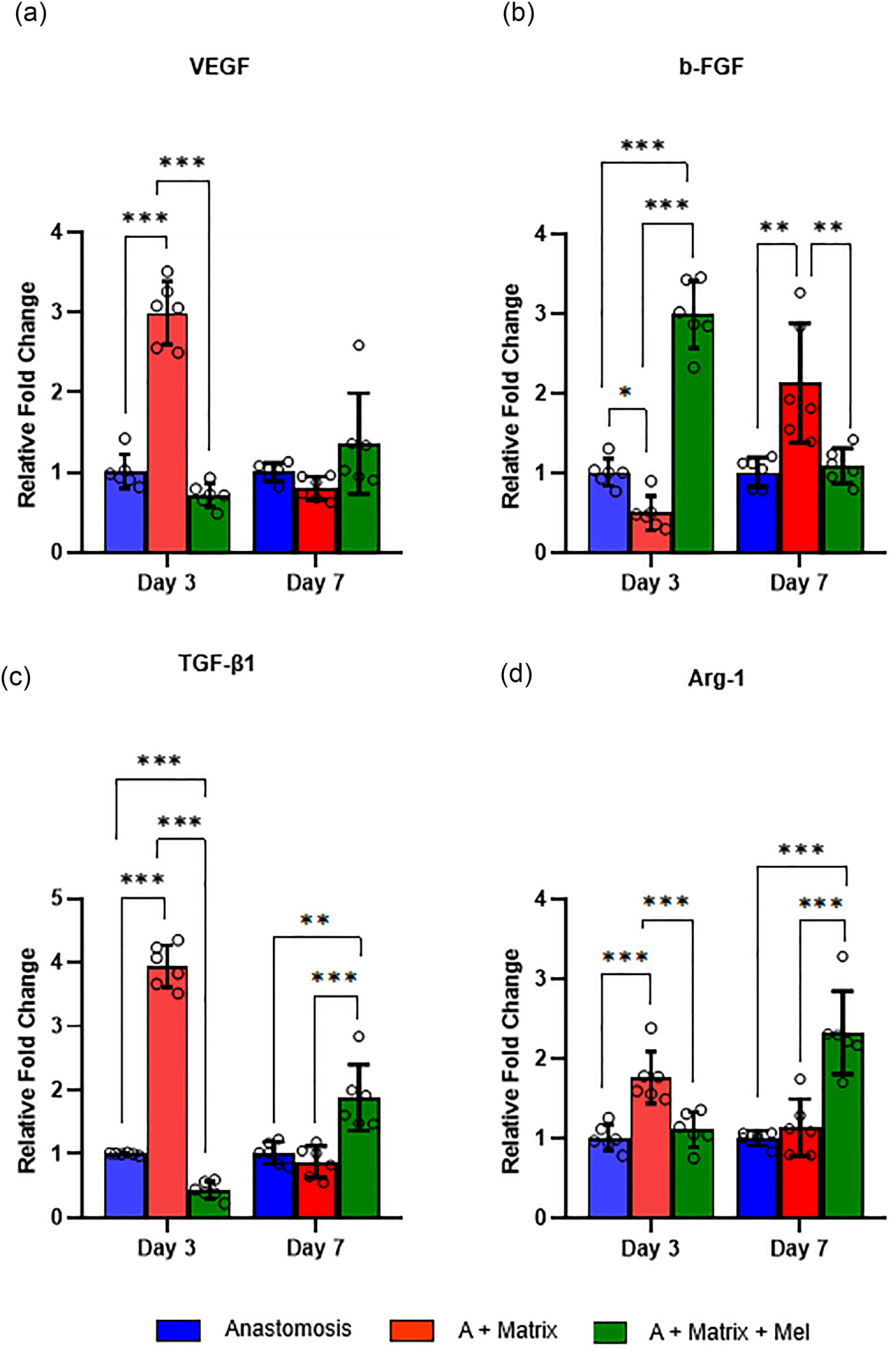
Relative mRNA expression of growth factors and the macrophage polarization marker in colonic anastomotic tissues at postoperative days 3 and 7. Bar graphs with overlaid individual data points represent the relative mRNA expression of the following: (a) *VEGF*; (b) *b‐FGF*; (c) *TGF‐β1*; and (d) *Arg‐1*. Data are presented as the mean ± SD (*n* = 6 animals per experimental group at each time point). Asterisks indicate statistically significant differences between the indicated experimental groups (*P ≤ 0.05, **P ≤ 0.01 and ***P ≤ 0.001).

The expression of growth factors and the macrophage polarization marker varied significantly among the experimental groups during the healing process (Figure 4a–d).

At POD 3, *VEGF* expression was significantly higher in the A+Matrix group than in both the A (*P* < 0.0001) and A+Matrix+Mel (*P* < 0.0001) groups, whereas no significant difference was detected between the A and A+Matrix+Mel groups (*P* = 0.177). By POD 7, *VEGF* expression was comparable among all groups, with no statistically significant differences observed (A vs. A+Matrix, *P* = 0.640; A vs. A+Matrix+Mel, *P* = 0.267; A+Matrix vs. A+Matrix+Mel, *P* = 0.056).

For *b‐FGF*, significant differences were detected among all three experimental groups at POD 3. The A+Matrix group exhibited significantly lower expression than the A group (*P* = 0.021), whereas the A+Matrix+Mel group demonstrated significantly higher expression than both the A (*P* < 0.0001) and A+Matrix (*P* < 0.0001) groups. At POD 7, *b‐FGF* expression remained significantly higher in the A+Matrix group than in both the A (*P* = 0.002) and A+Matrix+Mel (*P* = 0.004) groups, whereas no significant difference was detected between the A and A+Matrix+Mel groups (*P* = 0.954).

At POD 3, *TGF‐β1* expression was significantly higher in the A+Matrix group than in both the A (*P* < 0.0001) and A+Matrix+Mel (*P* < 0.0001) groups. In addition, the A group exhibited significantly higher *TGF‐β1* expression than the A+Matrix+Mel group (*P* = 0.0006). By POD 7, *TGF‐β1* expression was significantly higher in the A+Matrix+Mel group than in both the A (*P* = 0.0015) and A+Matrix (*P* = 0.0004) groups, whereas no significant difference was observed between the A and A+Matrix groups (*P* = 0.782).

Regarding *Arg‐1*, the A+Matrix group exhibited significantly higher expression than both the A (*P* = 0.0002) and A+Matrix+Mel (*P* = 0.0009) groups at POD 3, whereas no significant difference was detected between the A and A+Matrix+Mel groups (*P* = 0.771). At POD 7, *Arg‐1* expression was significantly higher in the A+Matrix+Mel group than in both the A (*P* = 0.000044) and A+Matrix (*P* = 0.000135) groups, whereas the latter two groups did not differ significantly (*P* = 0.808).

### 3.6 Matrix synthesis, contractile phenotype and protease activity

**FIGURE 5:**
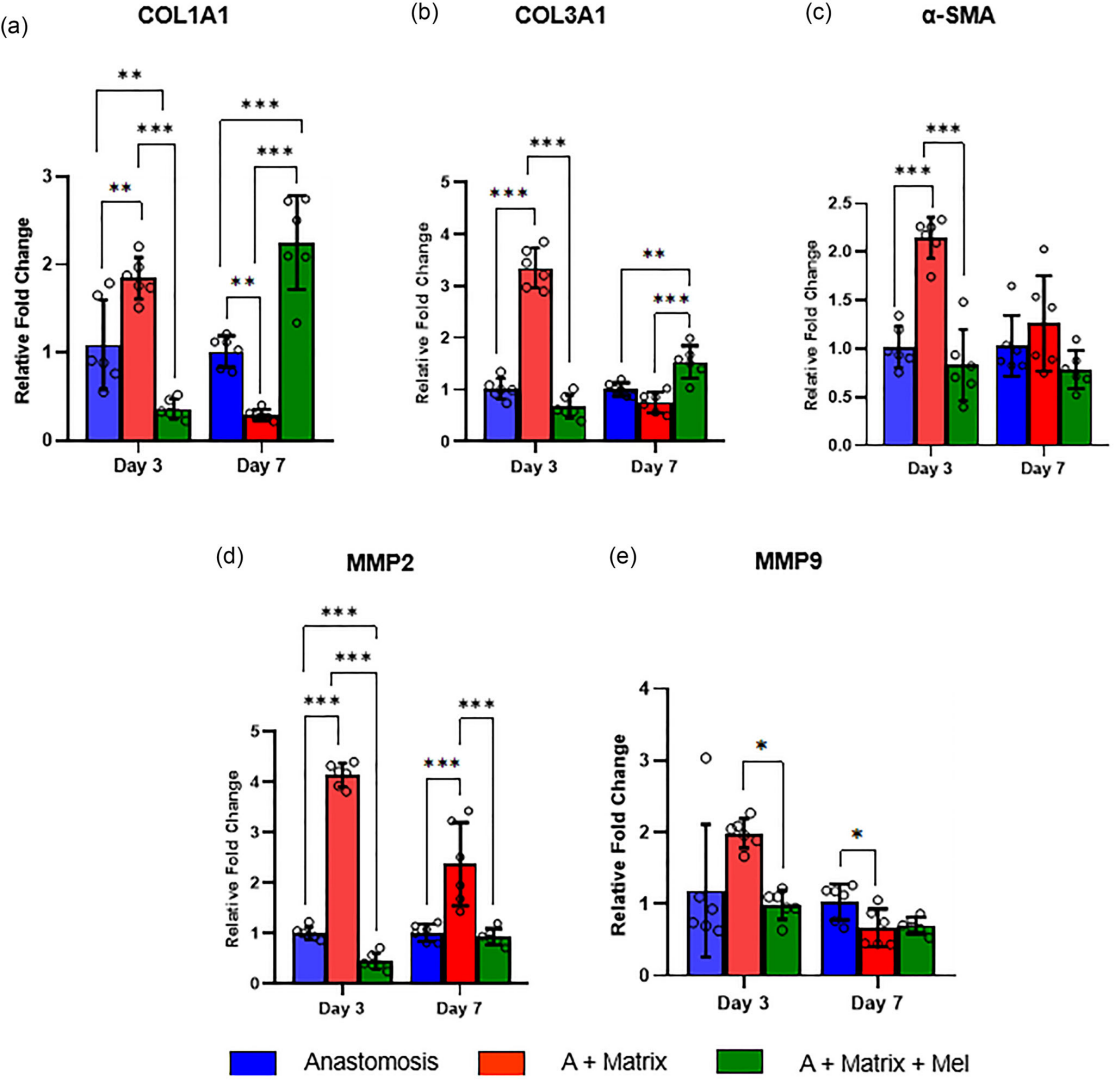
Relative mRNA expression of extracellular matrix components and matrix remodelling‐related genes in colonic anastomotic tissues at postoperative days 3 and 7. Bar graphs with overlaid individual data points represent the relative mRNA expression of the following: (a) *COL1A1*; (b) *COL3A1*; (c) *α‐SMA*; (d) *MMP2*; and (e) *MMP9*. Data are presented as the mean ± SD (*n* = 6 animals per experimental group at each time point). Asterisks indicate statistically significant differences between the indicated experimental groups (*P ≤ 0.05, **P ≤ 0.01 and ***P ≤ 0.001).

**FIGURE 6:**
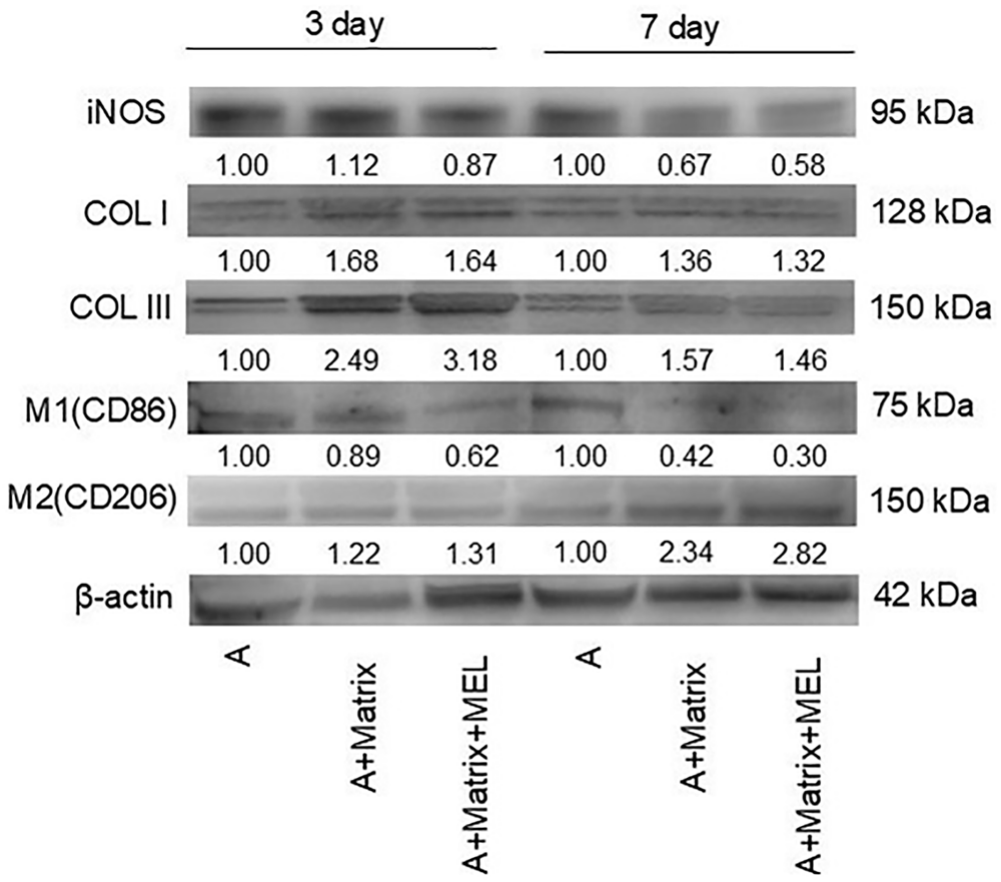
Western blot analysis of macrophage polarization and extracellular matrix protein expression at 3 and 7 days post‐treatment. Representative immunoblot images of pro‐inflammatory/M1 macrophage markers (iNOS and CD86), an anti‐inflammatory/M2 macrophage marker (CD206) and collagen deposition markers (COLI and COLIII). The experimental groups are as follows: A (control), A+Matrix, and A+Matrix+MEL (melatonin‐loaded matrix). β‐Actin served as the internal loading control. The numerical values beneath the bands indicate the relative densitometric fold change normalized to β‐actin, with the respective control group (A) set to 1.00 for each time point.

The expression of extracellular matrix‐related genes and matrix metalloproteinases varied significantly among the experimental groups during the healing process (Figure 5a–e). Additionally, Western blot analysis revealed significant alterations in the protein expression levels of iNOS, COL I, COL III, M1 (CD86), and M2 (CD206) among the experimental groups (Figure 6).

At POD 3, *COL1A1* expression was significantly higher in the A+Matrix group than in both the A (*P* = 0.003) and A+Matrix+Mel (*P* < 0.0001) groups. In addition, the A group exhibited significantly higher *COL1A1* expression than the A+Matrix+Mel group (*P* = 0.004). By POD 7, *COL1A1* expression was significantly higher in the A+Matrix +Mel group than in both the A (*P* < 0.0001) and A+Matrix (*P* < 0.0001) groups, whereas the A group also showed significantly higher expression than the A+Matrix group (*P* = 0.004).

For *COL3A1*, the A+Matrix group demonstrated significantly higher expression than both the A (*P* < 0.0001) and A+Matrix+Mel (*P* < 0.0001) groups at POD 3, whereas no significant difference was detected between the A and A+Matrix+Mel groups (*P* = 0.125). At POD 7, *COL3A1* expression was significantly increased in the A+Matrix+Mel group compared with both the A (*P* = 0.003) and A+Matrix (*P* < 0.0001) groups, whereas no significant difference was detected between the A and A+Matrix groups (*P* = 0.172).

At POD 3, α‐*SMA* expression was significantly higher in the A+Matrix group than in both the A (*P* < 0.0001) and A+Matrix+Mel (*P* < 0.0001) groups, whereas no significant difference was observed between the A and A+Matrix+Mel groups (*P* = 0.482). No statistically significant differences in α *‐SMA* expression were observed among the experimental groups at POD 7 (A vs. A+Matrix, *P* = 0.518; A vs. A+Matrix+Mel, *P* = 0.461; A+Matrix vs. A+Matrix+Mel, *P* = 0.081).

For *MMP2*, the A+Matrix group exhibited significantly higher expression than both the A (*P* < 0.0001) and A+Matrix+Mel (*P* < 0.0001) groups at POD 3. At POD 7, *MMP2* expression remained significantly higher in the A+Matrix group than in both the A (*P* = 0.0007) and A+Matrix+Mel (*P* = 0.0004) groups. In addition, the A group showed significantly higher *MMP2* expression than the A+Matrix+Mel group at POD 3 (*P* = 0.0002), whereas no significant difference between these two groups was detected at POD 7 (*P* = 0.960).

Regarding *MMP9*, the A+Matrix group demonstrated significantly higher expression than the A+Matrix+Mel group at POD 3 (*P* = 0.019), whereas other comparisons on this day were not significant. At POD 7, *MMP9* expression was significantly higher in the A group than in the A+Matrix group (*P* = 0.031), whereas no other pairwise comparisons reached statistical significance (A vs. A+Matrix+Mel, *P* = 0.051; A+Matrix vs. A+Matrix+Mel, *P* = 0.961).

#### 3.6.1 Western blot findings

Protein expression of COLI, COLIII, iNOS, CD86 and CD206 was assessed on days 3 and 7 in A, A+Matrix and A+Matrix+Mel groups. Overall, the western blot patterns were consistent with the gene‐expression trends. iNOS increased in A+Matrix versus A at day 3, and was lower in A+Matrix+Mel versus both groups at day 3; at day 7, A+Matrix+Mel remained the lowest. COLI was higher in A+Matrix and A+Matrix+Mel than A at both time points, and values were broadly similar between the two matrix groups. COLIII was elevated in both matrix groups versus A at days 3 and 7, with a stronger elevation at day 3; A+Matrix+Mel exceeded A+Matrix at day 3, and at day 7 the two matrix groups appeared closer. CD86 was lower in both matrix groups versus A at both time points, with a further reduction in A+Matrix+Mel relative to A+Matrix. CD206 was higher in both matrix groups versus A at both time points; A+Matrix+Mel exceeded A+Matrix at both day 3 and day 7, and the increase at day 7 was more pronounced than at day 3.

#### 3.6.2 Histopathological evaluation

**FIGURE 7:**
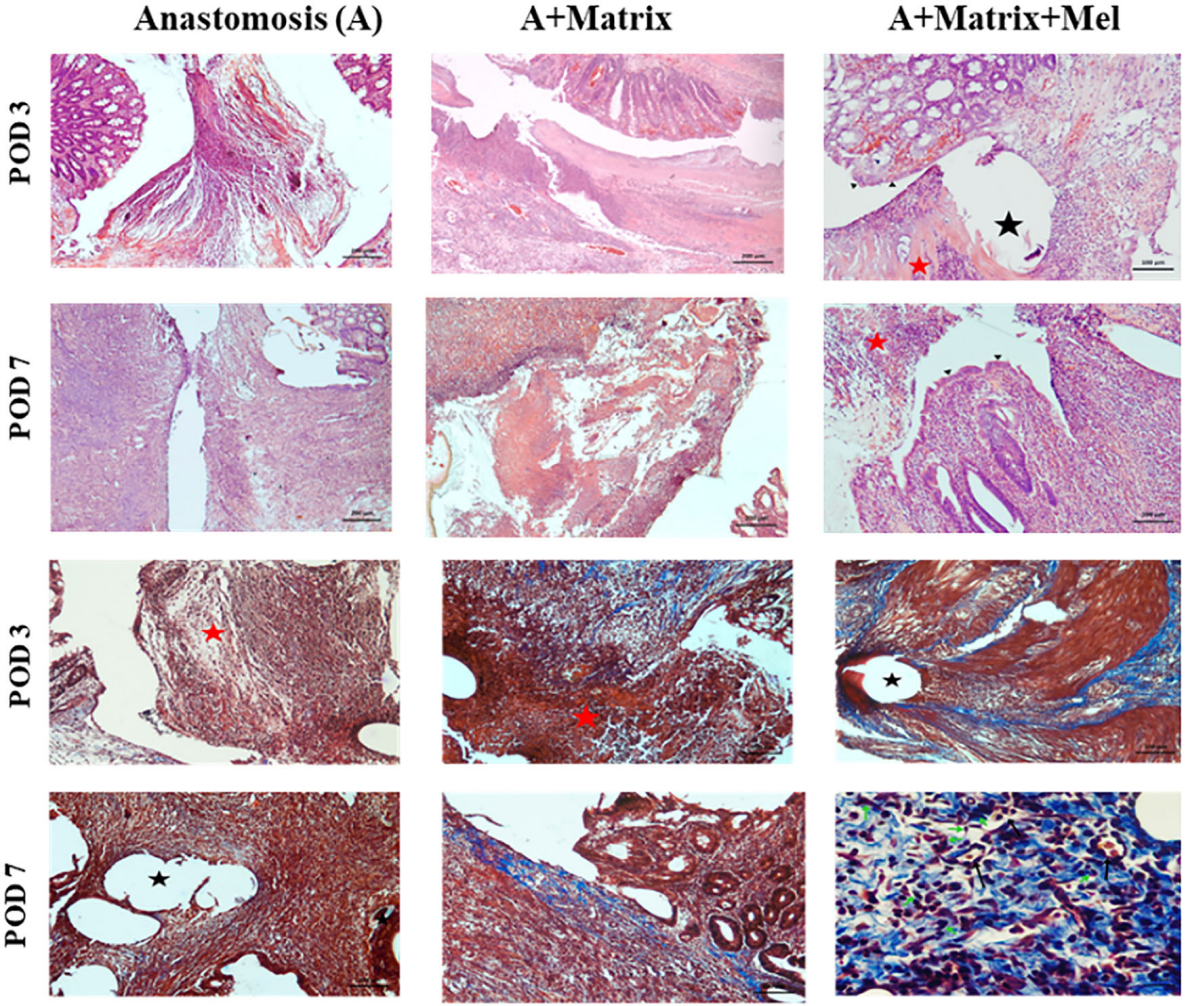
Representative histological images of the anastomotic tissues stained with H&E (top two rows) for evaluating general morphology and inflammation, and with MT (bottom two rows) for assessing collagen deposition. The images display the healing progression across the anastomosis (A), A+Matrix and A+Matrix+Mel groups. Black stars indicate the suture area; red stars denote granulation tissue, inflammatory cells. Abbreviations: H&E, Haematoxylin and Eosin; MT, Masson's Trichrome; POD, postoperative day.

**FIGURE 8:**
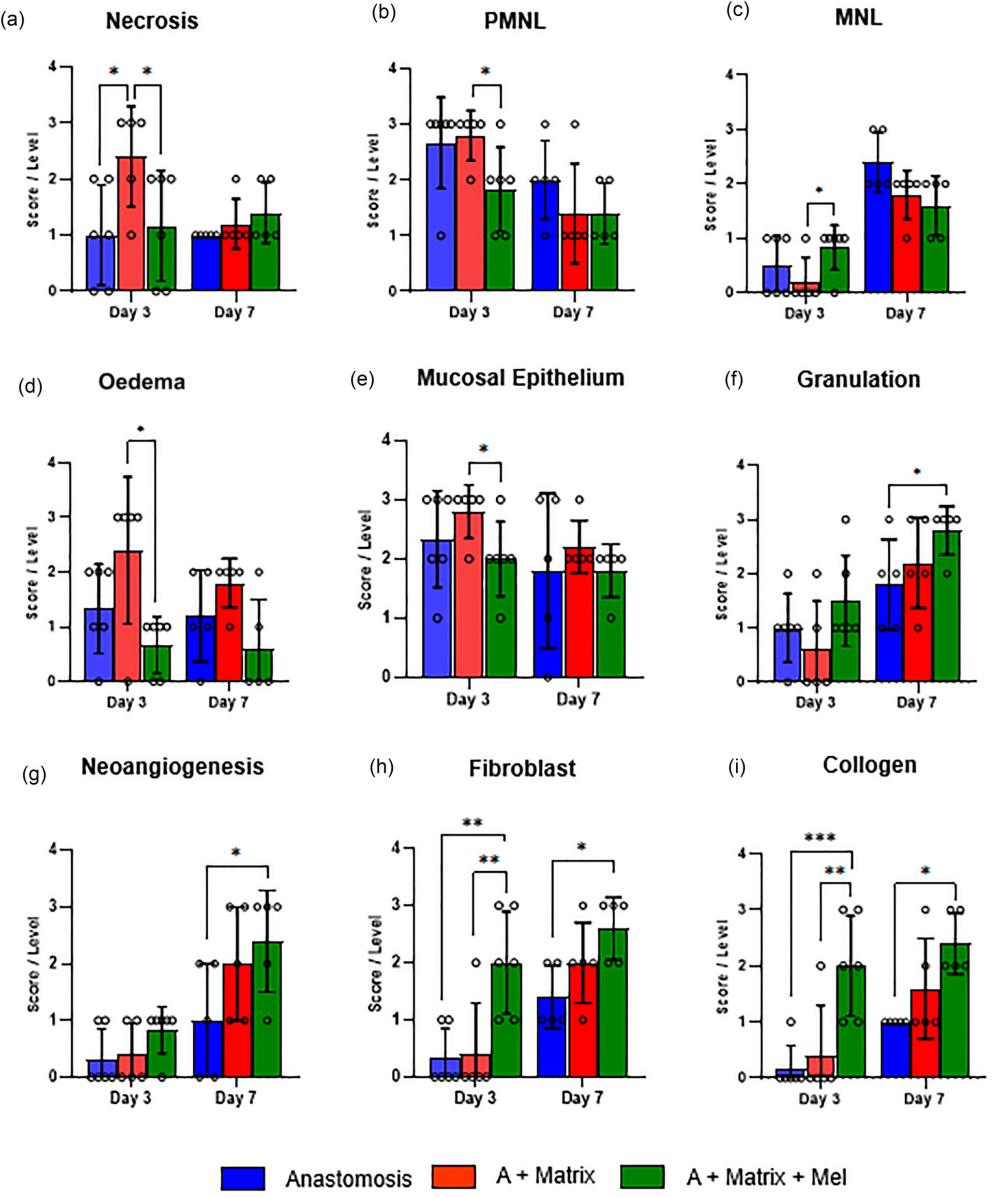
Semi‐quantitative histopathological assessment of colonic anastomotic tissues at postoperative days 3 and 7. Bar graphs with overlaid individual data points represent the histopathological scores for the following: (a) necrosis; (b) PMNL infiltration; (c) MNL infiltration; (d) oedema; (e) mucosal epithelium; (f) granulation tissue formation; (g) neoangiogenesis; (h) fibroblast proliferation; and (i) collagen deposition. Data are presented as the mean ± SD (*n* = 6 animals per experimental group at each time point). Asterisks indicate statistically significant differences (*P ≤ 0.05, **P ≤ 0.01 and ***P ≤ 0.001). Abbreviations: MNL, mononuclear leucocyte; PMNL, polymorphonuclear leucocyte.

Histological healing at the anastomotic site was assessed using Haematoxylin and Eosin staining with semi‐quantitative scoring of necrosis, inflammatory infiltrates (PMNL and MNL), oedema, integrity of the mucosal epithelium, granulation tissue, neoangiogenesis and fibroblast density. Collagen deposition was evaluated by Masson's Trichrome staining (Figures 7 and 8).

At POD 3, the A+Matrix group exhibited a less favourable acute histological profile, characterized by significantly higher necrosis than the A group (*P* = 0.038). Necrosis was significantly reduced in the A+Matrix+Mel group compared with the A+Matrix group (*P* = 0.050). Likewise, PMNL infiltration (*P* = 0.039), MNL infiltration (*P* = 0.045), oedema (*P* = 0.050) and mucosal epithelial injury (*P* = 0.044) were all significantly lower in the A+Matrix+Mel group than in the A+Matrix group. Fibroblast scores were significantly higher in the A+Matrix+Mel group than in both the A (*P* = 0.004) and A+Matrix (*P* = 0.010) groups, indicating enhanced early reparative cellular activity. Although formation of granulation tissue and neoangiogenesis were generally limited at this early stage, no statistically significant differences were observed among the groups.

By POD 7, oedema decreased in all groups, without significant differences among the treatment groups. Formation of granulation tissue and neoangiogenesis increased over time, with significantly higher scores in the A+Matrix+Mel group than in the A group (both *P* = 0.050). Fibroblast density also remained significantly higher in the A+Matrix+Mel group than in the A group (*P* = 0.020), consistent with an enhanced reparative response.

#### 3.6.3 Collagen deposition (Masson's Trichrome)

At POD 3, collagen deposition was limited in all groups but was significantly greater in the A+Matrix+Mel group than in both the A (*P* = 0.001) and A+Matrix (*P* = 0.009) groups. By POD 7, collagen deposition increased further, with the A+Matrix+Mel group demonstrating significantly greater collagen staining than the A group (*P* = 0.050). Within‐group comparisons showed a temporal increase in collagen deposition in the A and A+Matrix groups, whereas the increase observed in the A+Matrix+Mel group did not reach statistical significance.

## 4 DISCUSSION

In this study, we evaluated a PLGA–PEG200 electrospun nanofibrous matrix designed for local, controlled melatonin delivery in a rat colon anastomosis model. Overall, the findings indicate that sustained local melatonin release supports anastomotic repair, with the most evident effects observed at POD 7, a clinically relevant window when anastomoses remain vulnerable and leak diagnoses commonly emerge (Demir et al., 2022).

A key practical consideration was aligning material performance with the biology of early healing. The PLGA–PEG (70:30) matrix exhibited substantial degradation over 7 days (∼80% mass loss), consistent with the intended coverage of the critical early period. Release testing supported that melatonin remained available throughout this interval, and macroscopic observations at PODs 3 and 7 were compatible with ongoing presence of matrix at the anastomotic site. This local delivery strategy is particularly attractive because systemic melatonin often fails to maintain adequate, sustained concentrations in target tissues owing to rapid clearance and extensive first‐pass metabolism (Reiter et al., 2016; Tordjman et al., 2017). Accordingly, there is growing interest in biomaterial‐based, controlled‐release melatonin platforms (Ana González‐Cela‐Casamayor et al., 2025). To our knowledge, however, controlled melatonin delivery has not previously been assessed specifically in colon anastomotic healing, making the present work a focused contribution to this gap.

Functionally, the most direct readout of anastomotic integrity is bursting pressure, which reflects the mechanical competence of the healing intestinal wall and is strongly influenced by submucosal collagen architecture (Raptis et al., 2018). Consistent with prior experimental designs, we assessed bursting pressure at PODs 3 and 7 (Ozdemir et al., 2024). No between‐group differences were detected at POD 3, whereas POD 7 values were clearly higher in the melatonin‐matrix group compared with controls and the melatonin‐free matrix group. The rise in bursting pressure in the matrix‐only group suggests that mechanical reinforcement and/or biomaterial–tissue interactions can contribute to gains in strength even without drug loading. Still, the greater improvement observed in the melatonin‐loaded matrix group suggests that biochemical effects associated with melatonin might contribute to enhanced tissue repair beyond the structural support provided by the matrix alone.

These functional outcomes were accompanied by consistent molecular and histological patterns involving inflammation control, redox regulation and matrix remodelling. Anastomotic failure is closely linked to an imbalance between inflammatory injury, oxidative stress, matrix degradation and collagen synthesis during early healing. Locally delivered melatonin was associated with reduced expression of major pro‐inflammatory cytokines (IL‐6, IL‐1β and TNF‐α) and modulation of key inflammatory effectors (iNOS, COX‐2 and MPO) across PODs 3–7. This pattern is in line with the established anti‐inflammatory actions of melatonin across tissues (Minari & Pisani, 2025) and is relevant because persistently elevated cytokine signalling is known to amplify protease activity and impair extracellular matrix stability (Barrientos et al., 2008). Notably, wound nitric oxide dynamics are time dependent. Early NO can support perfusion, but sustained or excessive production, often reflected by higher iNOS, can promote chronic inflammation and compromise collagen assembly (Heo et al., 2025). The observed reduction in iNOS at POD 7 is therefore consistent with improved structural recovery.

Collagen deposition is a central determinant of tensile strength in intestinal repair, with type I and type III collagen being particularly important for restoring mechanical stability (Liu et al., 2022). In this study, the melatonin‐matrix group showed increased expression of *TGF‐β1*, *COL1A1* and *COL3A1*, together with histological evidence of higher collagen accumulation and a stronger fibroblast response. These findings align with the concept that timely activation of fibroblasts and balanced matrix production reduce leakage risk (Despoudi et al., 2025). Although reports on melatonin and collagen are not entirely consistent, some studies in pinealectomized rats have suggested reduced collagen synthesis (Bulbuller et al., 2005), whereas others report enhanced collagen deposition (Pugazhenthi et al., 2008). Differences in model context, dosing and route of administration are likely to contribute to heterogeneity. Our data support the view that local sustained delivery might shift the balance towards constructive remodelling, possibly through modulation of inflammatory nitric oxide pathways, as previously proposed (Pugazhenthi et al., 2008).

Extracellular matrix turnover is also governed by protease systems, such as MMP2 and MMP9, which participate in physiological remodelling but, when upregulated, can accelerate collagen degradation and destabilize the anastomosis. Elevated MMP activity has been linked to higher leakage rates in experimental settings (Neumann et al., 2018; Sunter et al., 2025). In our model, melatonin delivery was associated with reduced MMP2 and MMP9 expression at both time points, suggesting a more favourable remodelling environment. Additionally, we explored ADAM10 and ADAM17, proteases with broad roles in inflammatory signalling and tissue remodelling. Although their specific roles in colon anastomosis repair are not well characterized, the observed divergence of higher *ADAM10* and lower *ADAM17* at POD 7 in the melatonin group raises the possibility that melatonin treatment is associated with changes in remodelling‐related pathways not only through MMP pathways but also via ADAM‐mediated regulation of inflammatory signalling. This is compatible with reports suggesting links between melatonin and ADAM activity in other disease contexts (Elfiky et al., 2021), while emphasizing the need for targeted mechanistic work in intestinal repair models.

Macrophage polarization provides another integrative framework for interpreting early healing. Transition from an M1‐dominant inflammatory profile towards an M2‐like reparative phenotype supports resolution, collagen deposition and remodelling (Guo & DiPietro, 2010; Yunna et al., 2020). Arg‐1, a canonical marker linked to M2‐like activity, increased in the melatonin group, consistent with a reparative shift. Likewise, prior work in inflammatory bowel settings associates improved anastomotic outcomes with increased M2 signatures and Arg‐1 (Che et al., 2022; Kuninaka et al., 2022). Because Arg‐1 and iNOS compete for l‐arginine, an Arg‐1‐dominant milieu is often accompanied by reduced iNOS activity (Munder, 2009; Pourcet & Pineda‐Torra, 2013), a relationship that coheres with our POD 7 pattern and is consistent with a coordinated immunometabolic response.

Regarding oxidative stress markers, the interpretation deserves nuance. Several studies have linked improved anastomotic repair to increased antioxidant enzyme activity during certain interventions (Kandas et al., 2023; Özkan et al., 2018; Pehlivanlı et al., 2018). In our dataset, *CAT* and *HO‐1* were lower in the melatonin group, particularly at POD 7. This might reflect a reduced requirement for compensatory antioxidant responses owing to the direct radical‐scavenging capacity of melatonin and the stabilizing effect of sustained local exposure. In other words, lower expression of endogenous antioxidant enzymes in this setting might indicate attenuated oxidative burden, rather than impaired defence.

Finally, angiogenic and proliferative signalling is temporally dynamic and sensitive to hypoxia, inflammatory load and tissue perfusion (Ceccarelli et al., 2007), although the literature often shows broadly parallel trends of VEGF and FGF‐2 across wound phases (Duran et al., 2025; Yen et al., 2022). Early time points can be variable because multiple pathways are active simultaneously, and the kinetics of neovascularization and proliferation are not instantaneous. Thus, differences at POD 3 should be interpreted cautiously, whereas the histological evidence of enhanced neoangiogenesis at POD 7 supports a net pro‐repair effect at the tissue level.

### 4.1 Limitations

The present study has certain limitations that should be acknowledged. First, the biological evaluations were conducted exclusively on male rats to eliminate cyclical hormonal variations, which limits the direct extrapolation of these findings to female subjects. Second, although an extensive panel of parameters was analysed to characterize the healing process, this study was designed primarily to evaluate structural and functional outcomes rather than defining the definitive upstream molecular mechanisms of melatonin interactions. Third, the present protocol focused on a single optimization dose in a standardized healthy model without tracking systemic serum profiles. Further studies addressing varied clinical comorbidities and dose–response dynamics will be valuable to expand upon these baseline outcomes.

## 5 CONCLUSION

Taken together, this work suggests that a PLGA–PEG200 nanofibrous matrix can function as a dual‐action platform in colon anastomosis: mechanical reinforcement plus sustained local melatonin delivery. The results support a model in which controlled melatonin exposure improves the healing microenvironment by tempering inflammatory signalling (*IL‐6, IL‐1β, TNF‐α* and *iNOS/COX‐2/MPO*), reducing matrix‐degrading activity (MMP2/MMP9), promoting reparative polarization (*Arg‐1*) and enhancing collagen synthesis and remodelling (TGF‐β1, COL1A1, COL3A1 and ADAM10/ADAM17), which collectively align with higher collagen deposition and improved mechanical strength at POD 7. Further studies are warranted to test this approach in conditions of compromised healing, fully elucidate the underlying molecular mechanisms and delineate the distinct contribution of the matrix itself, given the measurable effects observed in the melatonin‐free PLGA–PEG group.

## AUTHOR CONTRIBUTIONS

Ufuk Demir: Methodology, investigation, writing—original draft. Zekiye Gülfem Yurtgezen: Formal analysis, data curation, visualization. Gülfem Erbil: Formal analysis, data curation, visualization, writing—review & editing. Oruç Numan Gökçe: Investigation, resources. Ayhan Oral: Methodology, validation, resources. Metehan Uzun: Conceptualization, supervision, project administration, funding acquisition, writing—review & editing. All authors approved the final version of the manuscript and agree to be accountable for all aspects of the work in ensuring that questions related to the accuracy or integrity of any part of the work are appropriately investigated and resolved. All persons designated as authors qualify for authorship, and all those who qualify for authorship are listed.

## CONFLICT OF INTEREST

The authors declare that they have no known competing financial interests or personal relationships that could have appeared to influence the work reported in this paper.

## GENERATIVE AI STATEMENT

During the preparation of this work, the authors used Gemini in order to improve the language and readability of the manuscript. After using this tool, the authors reviewed and edited the content as needed and take full responsibility for the content of the publication.

## Data Availability

The datasets generated and/or analysed during the present study are available from the corresponding author on reasonable request.
